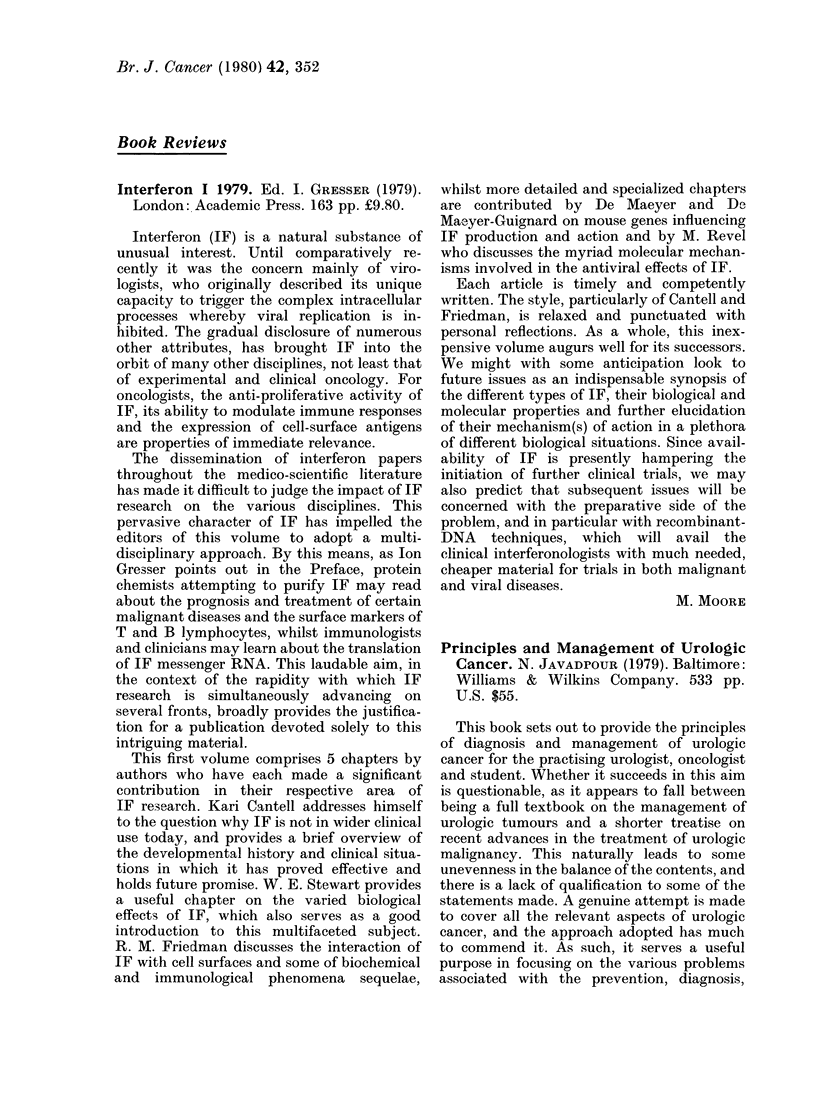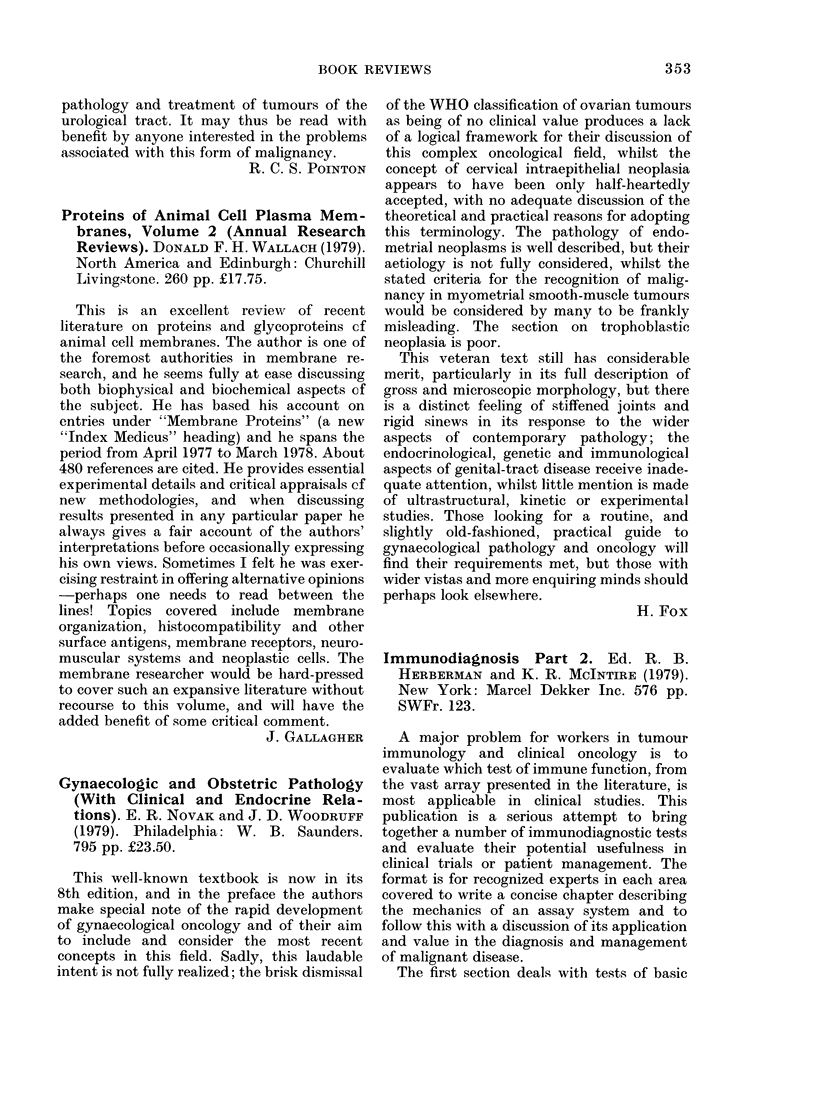# Principles and Management of Urologic Cancer

**Published:** 1980-08

**Authors:** R. C. S. Pointon


**Principles and Management of Urologic Cancer.** N. JAVADPOUR (1979). Baltimore: Williams & Wilkins Company. 533 pp. U.S. $55.

This book sets out to provide the principles of diagnosis and management of urologic cancer for the practising urologist, oncologist and student. Whether it succeeds in this aim is questionable, as it appears to fall between being a full textbook on the management of urologic tumours and a shorter treatise on recent advances in the treatment of urologic malignancy. This naturally leads to some unevenness in the balance of the contents, and there is a lack of qualification to some of the statements made. A genuine attempt is made to cover all the relevant aspects of urologic cancer, and the approach adopted has much to commend it. As such, it serves a useful purpose in focusing on the various problems associated with the prevention, diagnosis, pathology and treatment of tumours of the urological tract. It may thus be read with benefit by anyone interested in the problems associated with this form of malignancy.

R. C. S. Pointon